# *Bcl-xL* as a poor prognostic biomarker and predictor of response to adjuvant chemotherapy specifically in *BRAF*-mutant stage II and III colon cancer

**DOI:** 10.18632/oncotarget.24481

**Published:** 2018-02-13

**Authors:** Philip D. Dunne, Helen G. Coleman, Peter Bankhead, Matthew Alderdice, Ronan T. Gray, Stephen McQuaid, Victoria Bingham, Maurice B. Loughrey, Jacqueline A. James, Amy M.B. McCorry, Alan Gilmore, Caitriona Holohan, Dirk Klingbiel, Sabine Tejpar, Patrick G. Johnston, Darragh G. McArt, Federica Di Nicolantonio, Daniel B. Longley, Mark Lawler

**Affiliations:** ^1^ Centre for Cancer Research and Cell Biology, Queens’s University Belfast, Belfast, UK; ^2^ Centre for Public Health, Queens’s University Belfast, Belfast, UK; ^3^ SAKK Swiss Group for Clinical Cancer Research, Coordinating Center, Bern, Switzerland; ^4^ Digestive Oncology Unit, University Hospital Gasthuisberg, Leuven, Belgium; ^5^ University of Turin, Department of Oncology, Candiolo, Turin, Italy; ^6^ Candiolo Cancer Institute - FPO, IRCCS, Candiolo, Turin, Italy; ^7^ SIB Swiss Institute of Bioinformatics, Bioinformatics Core Facility, University of Lausanne, Lausanne, Switzerland

**Keywords:** colon cancer, gene expression profiling, molecular stratification, relapse risk, Bcl-xL

## Abstract

**Purpose:**

BRAF mutation occurs in 8–15% of colon cancers (CC), and is associated with poor prognosis in metastatic disease. Compared to wild-type BRAF (BRAFWT) disease, stage II/III CC patients with BRAF mutant (BRAFMT) tumors have shorter overall survival after relapse; however, time-to-relapse is not significantly different. The aim of this investigation was to identify, and validate, novel predictors of relapse of stage II/III BRAFMT CC.

**Experimental design:**

We used gene expression data from a cohort of 460 patients (GSE39582) to perform a supervised classification analysis based on risk-of-relapse within BRAFMT stage II/III CC, to identify transcriptomic biomarkers associated with prognosis within this genotype. These findings were validated using immunohistochemistry in an independent population-based cohort of Stage II/III CC (*n* = 691), applying Cox proportional hazards analysis to determine associations with survival.

**Results:**

High gene expression levels of Bcl-xL, a key regulator of apoptosis, were associated with increased risk of relapse, specifically in BRAFMT tumors (HR = 8.3, 95% CI 1.7–41.7), but not KRASMT/BRAFWT or KRASWT/BRAFWT tumors. High Bcl-xL protein expression in BRAFMT, untreated, stage II/III CC was confirmed to be associated with an increased risk of death in an independent cohort (HR = 12.13, 95% CI 2.49–59.13). Additionally, BRAFMT tumors with high levels of Bcl-xL protein expression appeared to benefit from adjuvant chemotherapy (P for interaction = 0.006), indicating the potential predictive value of Bcl-xL expression in this setting.

**Conclusions:**

These findings provide evidence that Bcl-xL gene and/or protein expression identifies a poor prognostic subgroup of BRAFMT stage II/III CC patients, who may benefit from adjuvant chemotherapy.

## INTRODUCTION

Signaling through the Epidermal Growth Factor Receptor (EGFR) pathway is a common event in cancer development [1], with activating mutations in *KRAS, NRAS* and *BRAF* occurring in approximately 50% of colorectal cancer (CRC) patients [2]. Results from a phase III trial (MRC COIN trial, *n* = 1630) in metastatic CRC revealed that patients with *BRAF* mutant (*BRAFMT)* tumors have a significantly worse prognosis compared to patients with *KRAS* mutant (*KRASMT)* tumors or tumors with no detectable mutations in *KRAS* or *BRAF* (*WT/WT*) [3]. The use of a *BRAFMT* specific inhibitor, vemurafenib, in advanced melanoma, has improved survival rates for patients with this activating mutation [4], and underpinned the rationale for a phase Ib study employing vemurafenib in *BRAFMT* CRC [5]. Unfortunately, unlike the encouraging results observed in *BRAFMT* melanoma, the inhibitor did not benefit *BRAFMT* CRC patients in the advanced disease setting. Mechanistic studies have indicated that resistance to vemurafenib in CRC is due to feedback activation of the EGFR pathway [6], further highlighting the key role played by EGFR signaling in CRC.

To examine the role of *BRAF* in the adjuvant stage II/III disease setting, Popovici and colleagues performed differential gene expression analysis to identify transcriptional differences between *BRAFMT* and *BRAFWT/KRASWT* tumors in a cohort of 688 stage II and III colon cancer (CC) clinical trial samples (PETACC-3) [7]. Their analysis identified the distinct underlying biology of the *BRAFMT* subgroup. Furthermore, the authors generated a 64-gene classifier, which stratified the cohort into two subgroups. The first subgroup, which accounted for 27% of the cohort, displayed a transcriptional signature similar to *BRAFMT* tumors (termed “pred-BRAFm”) and had a worse prognosis in terms of overall survival (OS) and survival-after-relapse compared to the second subgroup, which had a signature similar to that of *BRAFWT* disease (termed “pred-BRAFwt”). Critically however, while both *BRAF* mutation and the pred-BRAFm signatures could identify subgroups of patients with poorer OS *after* relapse (i.e. when the patient had progressed to stage IV metastatic disease), the rates of disease relapse in these subgroups were not significantly different to *BRAFWT* and pred-BRAFwt disease.

There is currently a lack of understanding of the biology that drives disease relapse specifically within stage II/III *BRAFMT* disease, resolution of which could ultimately inform treatment of a clinically-definable subgroup of *BRAFMT* patients, who have the worst prognosis when they progress to stage IV, but who still may be potentially curable in stage II/III. Therefore, we aimed to identify novel predictors of relapse for stage II/III *BRAFMT* CC, employing transcriptomic datasets for *in silico* discovery/initial corroboration, followed by subsequent validation of promising lead candidate(s) from bioinformatics analyses by immunohistochemistry analysis within a large population-based stage II/III *BRAFMT* CC study.

## RESULTS

### Study outline and rationale for risk stratification in BRAFMT CC

We analyzed available transcriptional data from the well-characterized dataset, GSE39582, as outlined in Supplementary Figure 1. Compared to *KRASMT* and *WT/WT* patients, *BRAFMT* patients were significantly more likely to be older (*p* < 0.001), have proximal tumors (*p* < 0.001) that exhibited microsatellite instability (MSI, *p* < 0.001) and to be assigned as Consensus Molecular Subtype 1 (CMS1, *p* < 0.001) (Table 1). Additionally, patients with *BRAFMT* tumors were significantly more likely to be female (*p* = 0.04 and *p* = 0.001) and to receive no adjuvant chemotherapy (*p* = 0.001 and *p* = 0.006) compared to *KRASMT* and *WT/WT* respectively (Table 1). Finally, *BRAFMT* patients were significantly more likely to have later stage disease (stage II v III) compared to *WT/WT* patients (*p* = 0.04) (Table 1). Using the 64 gene *BRAF* classifier identified by Popovici *et al.* [7] we performed semi-supervised hierarchical clustering of the gene expression profiles of the entire stage II/III patient cohort. We identified a subgroup accounting for 28% (*n* = 127) of the tumor profiles using this method of clustering, which displayed an expression pattern similar to the pred-BRAFm profile (Supplementary Figure 2A). We found no difference in relapse rates between the pred-BRAFm and the pred-BRAFwt populations in this cohort (Supplementary Figure 2A; HR = 0.95 (95% CI 0.65–1.39)).

**Table 1:** Characteristics of colon cancer patients and tumors according to BRAF and KRAS status.

Characteristic	BRAF MT *n* = 41	KRAS MT *n* = 166	*p*-value	WT/WT *n* = 210	*p*-value
**Age, years, mean (SD)**	76.0 (7.3)	67.7 (13.5)	<0.001	65.6 (12.6)	<0.001
**Sex, *n* (%)** Male Female	14 (34.1) 27 (65.9)	86 (51.8) 80 (48.2)	0.04	130 (61.9) 80 (38.1)	0.001
**Tumour stage, *n* (%)** II III	20 (48.8) 21 (51.2)	86 (51.8) 80 (48.2)	0.73	138 (65.7) 72 (34.3)	0.04
**Tumour location, *n* (%)** Proximal Distal	37 (90.2) 4 (9.8)	86 (51.8) 80 (48.2)	<0.001	49 (23.3) 161 (76.1)	<0.001
Adjuvant treatment^*^ **receipt, *n* (%)** No Yes	33 (80.5) 8 (19.5)	86 (51.8) 80 (48.2)	0.001	121 (57.6) 89 (42.4)	0.006
**MSI status** MSI MSS Unknown	27 (65.9) 8 (19.5) 6 (14.6)	15 (9.0) 138 (83.1) 13 (7.8)	<0.001	15 (7.1) 170 (81.0) 25 (11.9)	<0.001
**Consensus Molecular Subtype, *n* (%)** CMS 1 CMS 2 CMS 3 CMS 4 Unknown	32 (78.1) 0 (0.0) 3 (7.3) 3 (7.3) 3 (7.3)	17 (10.2) 53 (31.9) 35 (21.1) 45 (27.1) 16 (9.6)	<0.001	26 (12.4) 120 (57.1) 9 (4.3) 40 (19.1) 15 (7.1)	<0.001

Characteristics of colon cancer patients and tumours according to BRAF and KRAS status.

MSI: Microsatellite instability; MSS: Microsatellite stable; MT: Mutant; WT/WT: BRAF and KRAS wild-type. ^*^Adjuvant chemotherapy treatment receipt.

Of the 460 tumor profiles within our cohort, 417 have KRAS and BRAF mutational analysis data. Comparative analysis was performed for age, sex, stage, location, treatment, MSI and Consensus Molecular Subtype (CMS). MSI: Microsatellite instability; MSS: Microsatellite stable; MT: Mutant; WT/WT: BRAF and KRAS wild-type. ^*^Adjuvant chemotherapy treatment received.

### Gene expression associated with risk of relapse in BRAFMT CC

Gene Set enrichment analysis (GSEA) of the discovery subset indicated increased myogenesis, epithelial-to-mesenchymal transition (EMT) and hypoxia pathways in the *BRAFMT* tumors with the highest-risk of disease relapse (Supplementary Figure 2B). Additionally, using the Microenvironment Cell Populations-counter (MCP), we identified a non-significant trend for increased fibroblasts in high-risk *BRAFMT* tumors (Supplementary Figure 2C). Using differential gene expression analysis contrasting profiles from high-risk or low-risk *BRAFMT* tumors in the discovery subset (Supplementary Figure 1), we identified 83 probesets (Supplementary Table 1) corresponding to 67 annotated genes that are prognostic for relapse risk in *BRAFMT* tumors; high expression of 43 genes were associated with increased risk of relapse, and high expression of 24 genes with decreased risk of relapse (Table 2). Increased expression of endoplasmic reticulum stress-induced transcripts such as PPP1R15A (GADD34), heat shock proteins HSPA6 and DNAJB1, and the stress-related transcription factor DDIT3 were observed in *BRAFMT* tumours with the highest-risk of disease relapse.

**Table 2:** Gene list associated with relapse in *BRAFMT* tumors

Symbol	Entrez Gene Name	Symbol	Entrez Gene Name
AEBP1	AE binding protein 1	AGR2	anterior gradient 2, protein disulphide isomerase family member
ALPP	alkaline phosphatase, placental	C2orf72	chromosome 2 open reading frame 72
ANGPTL1	angiopoietin-like 1	C3orf70	chromosome 3 open reading frame 70
BCL2L1	BCL2-like 1	COBL	cordon-bleu WH2 repeat protein
CCDC71L	coiled-coil domain containing 71-like	EFNA2	ephrin-A2
CCL7	chemokine (C-C motif) ligand 7	GATA6-AS1	GATA6 antisense RNA 1 (head to head)
CDA	cytidine deaminase	GMDS	GDP-mannose 4,6-dehydratase
CYSRT1	cysteine-rich tail protein 1	HES6	hes family bHLH transcription factor 6
DDIT3	DNA-damage-inducible transcript 3	IMPA2	inositol(myo)-1(or 4)-monophosphatase 2
DNAJB1	DnaJ (Hsp40) homolog, subfamily B, member 1	KIAA1324	KIAA1324
DNTTIP1	deoxynucleotidyltransferase, terminal, interacting protein 1	KIAA1671	KIAA1671
DUSP14	dual specificity phosphatase 14	KREMEN1	kringle containing transmembrane protein 1
EPYC	epiphycan	LARGE	like-glycosyltransferase
FST	follistatin	NOX1	NADPH oxidase 1
FXYD5	FXYD domain containing ion transport regulator 5	NRARP	NOTCH-regulated ankyrin repeat protein
GAS1	growth arrest-specific 1	PER2	period circadian clock 2
GJB3	gap junction protein, beta 3, 31kDa	PIP5K1B	phosphatidylinositol-4-phosphate 5-kinase, type I, beta
GJB5	gap junction protein, beta 5, 31.1kDa	PSMG4	proteasome (prosome, macropain) assembly chaperone 4
HCFC1R1	host cell factor C1 regulator 1 (XPO1 dependent)	SKP2	S-phase kinase-associated protein 2, E3 ubiquitin protein ligase
HSPA6	heat shock 70kDa protein 6 (HSP70B’)	SLC22A23	solute carrier family 22, member 23
IER5L	immediate early response 5-like	SPRED2	sprouty-related, EVH1 domain containing 2
IGFBP6	insulin-like growth factor binding protein 6	TMEM30B	transmembrane protein 30B
KLK10	kallikrein-related peptidase 10	TRIM15	tripartite motif containing 15
KRT16	keratin 16, type I	TSPAN13	tetraspanin 13
MFGE8	milk fat globule-EGF factor 8 protein		
MIR100HG	mir-100-let-7a-2 cluster host gene		
MYH4	myosin, heavy chain 4, skeletal muscle		
NKIRAS1	NFKB inhibitor interacting Ras-like 1		
NPC1L1	NPC1-like 1		
NT5E	5’-nucleotidase, ecto (CD73)		
PAEP	progestagen-associated endometrial protein		
PDP1	pyruvate dehyrogenase phosphatase catalytic subunit 1		
PHLDA3	pleckstrin homology-like domain, family A, member 3		
PPP1R15A	protein phosphatase 1, regulatory subunit 15A		
PRR9	proline rich 9		
RBMS2	RNA binding motif, single stranded interacting protein 2		
RGS4	regulator of G-protein signaling 4		
TAGLN3	transgelin 3		
TGFB2	transforming growth factor, beta 2		
TNFSF4	tumor necrosis factor (ligand) superfamily, member 4		
VEGFB	vascular endothelial growth factor B		
ZFAS1	ZNFX1 antisense RNA 1		
ZNF667-AS1	ZNF667 antisense RNA 1 (head to head)		

Genes associated with increased (red) and decreased (green) relapse risk from our BRAFMT risk-supervised differential gene expression analysis data.

**Figure 1:**
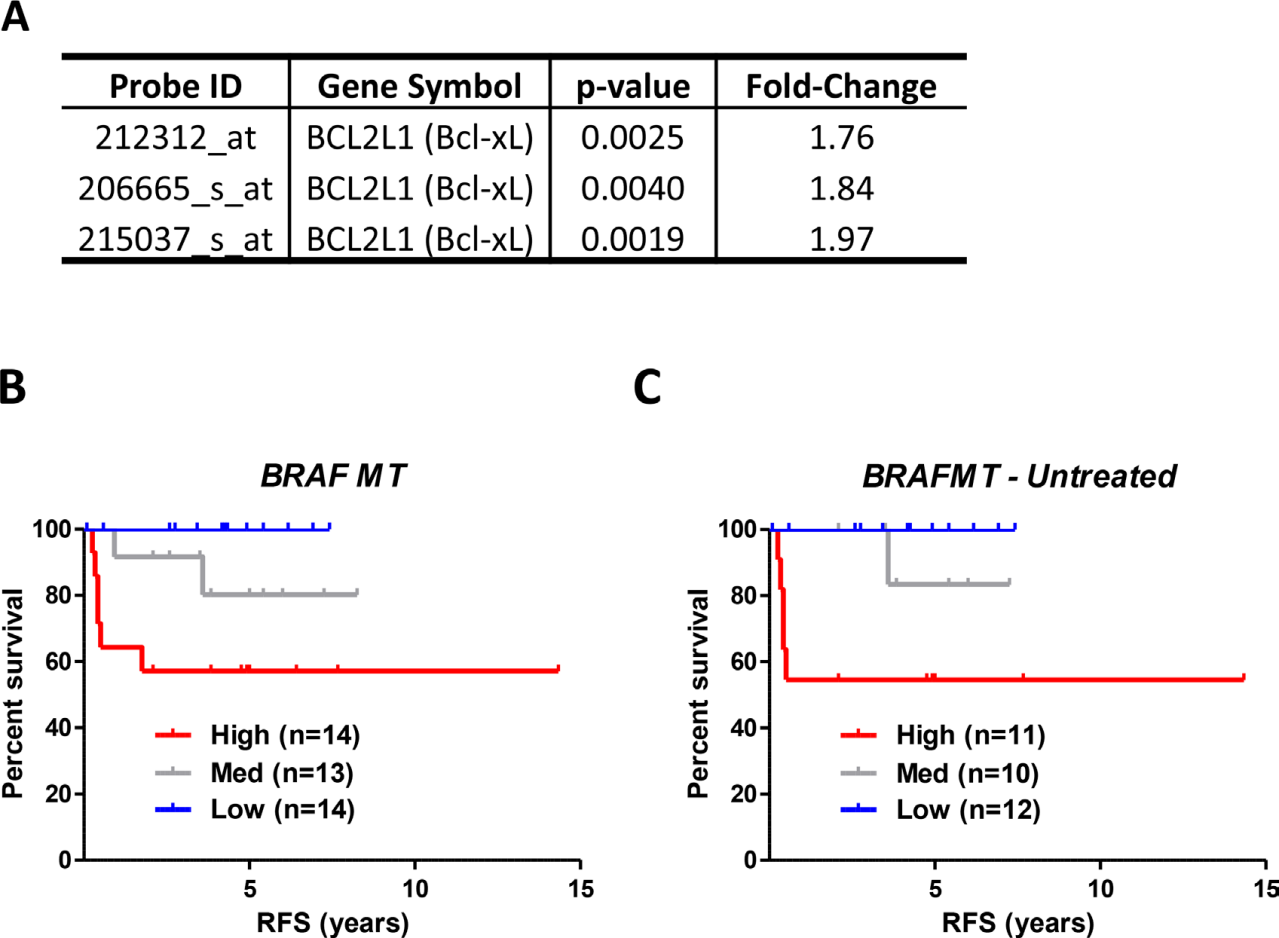
Relapse risk analysis of *BRAFMT* tumors indicates that *Bcl-xL* gene expression is associated with prognosis in *BRAFMT* tumors (**A**) BCL2L1 (*Bcl-xL*) was represented by 3 individual probesets in relapse risk analysis in *BRAFMT* tumors. (**B** and **C**) Relapse-free survival (RFS) curve using Kaplan-Meier estimation in the “Initial Consolidation” dataset comparing tertile stratified *Bcl-xL* gene expression levels in all *BRAFMT* (A) and untreated *BRAFMT* (B) stage II/III CRC patients. Unadjusted and adjusted HR statistics are detailed in Table 3.

While the majority of the 67 genes are represented by a single probeset, *BCL2L1* (encoding *Bcl-xL*) and *NCRNA00275* (which transcribes *ZFAS1*) are both represented by 3 *different* probesets (of the 4 total probesets for each gene), reducing the probability of the single genes themselves being false positives, which could potentially confound the validity of genes identified by a single probeset only (Supplementary Table 1). Gene expression levels of *Bcl-xL* were increased between 1.76–1.97 fold (Figure 1A) and *ZFAS1* by 1.83–1.90 fold (Supplementary Table 1) in the high-risk group compared to the low risk group. Importantly, the 67 *BRAFMT* prognostic gene list is distinct from the pred-BRAFm classifier reported by Popovici, as only one gene, (Kallikrein-Related Peptidase 10 (KLK10)), overlaps between these 2 gene lists (Supplementary Figure 2D).

### Probesets associated with risk in BRAFMT tumors represent distinct novel prognostic biology

As *BRAF* and *KRAS* are both key components of the EGFR/MAPK pathway, we performed a similar risk association analysis in *KRASMT* tumors and identified 139 probesets associated with risk-of-relapse in this genetic subgroup (Supplementary Table 2). We found no overlap between the probesets associated with risk-of-relapse in the *KRASMT* subgroup and the probesets identified in the *BRAFMT* analyses (Supplementary Figure 2E), indicating that distinct biologies determine prognosis in these two subgroups, at least in stage II/III disease.

### Bcl-xL mRNA expression is associated with poor prognosis in stage II/III BRAFMT CC

To confirm the clinical relevance of elevated *Bcl-xL* gene expression in our training set, in addition to testing the genotype-specific nature of its prognostic value, we next generated an “Initial Consolidation” dataset (*n* = 417, Supplementary Figure 1) by removing the filters initially applied to the discovery subset of the GSE39582 cohort, (i.e. we removed the restrictions on chemotherapy treatment and the follow-up criteria as detailed in Methods). This set of 417 patients represents an ideal cohort to assess the prognostic value of *Bcl-xL* in *KRASWT* and *WT/WT* patients that were not used to identify *Bcl-xL,* in addition to a further 17 *BRAFMT* patients that were previously excluded from the discovery data. Within *BRAFMT* tumors (*n* = 41), the *Bcl-xL*-high group (*Bcl-xL*^high^) had a significantly higher risk-of-relapse compared to the *Bcl-xL*-low (*Bcl-xL*^low^) expression group, using either an unadjusted (HR = 5.83), or adjusted model (HR = 9.63) accounting for potential confounding factors including age, gender, TNM stage and MSI status (confidence intervals could not be calculated due to an absence of events in *Bcl-xL*^low^; Figure 1B and Table 3). When examining untreated patients only, the prognostic value of *Bcl-xL* mRNA expression in *BRAFMT* patients was again apparent (Figure 1C); however, the prognostic value of *Bcl-xL* in the chemotherapy-treated patient subgroup could not be evaluated due to small numbers (*n* = 8). The *Bcl-xL* medium expression group (*Bcl-xL*^med^) displayed an intermediate relapse profile compared to the *Bcl-xL*^high^ and *Bcl-xL*^low^, suggesting a dose-response association between relapse risk and *Bcl-xL* gene expression. Stratification based on the median also demonstrated the prognostic value of *Bcl-xL* gene expression (HR = 5.24 (95% CI 1.3–21.2)) (Supplementary Figure 3A).

**Table 3:** Unadjusted and adjusted analyses of relapse-free survival

Bcl-xL	Non-progressors *n*	Progressors *n*	Unadjusted Hazard ratios (95% confidence intervals)	Adjusted^**^ Hazard ratios (95% confidence intervals)
**BRAF MT**				
Low	14	0	1.00	1.00
Medium	11	2	1.80 (Not calculable)	3.94 (Not calculable)
High	8	6	5.83 (Not calculable)	9.63 (Not calculable)
**KRAS MT**				
Low	38	18	1.00	1.00
Medium	33	21	1.45 (0.75–2.77)	1.47 (0.76–2.84)
High	34	22	1.25 (0.65–2.40)	1.32 (0.68–2.56)
**WT/WT**				
Low	57	13	1.00	1.00
Medium	53	17	1.39 (0.67–2.85)	1.27 (0.61–2.64)
High	50	20	1.54 (0.77–3.10)	1.47 (0.72–3.01)

MT: Mutant; WT/WT: BRAF and KRAS wild-type.

^*^Cut-offs for low/medium/high Bcl-xl gene expression based on tertile values within each BRAF/KRAS status subgroup.

^**^Adjustments included age and sex, and were tested for TNM stage, MSI status, adjuvant chemotherapy receipt and tumour location for all models. A backwards elimination model was applied for tested confounders until all were significant at the *p* < 0.25 level in the model. Final adjustments included age, sex, TNM stage and MSI status (for BRAF MT and WT/WT); age, sex, TNM stage, adjuvant chemotherapy receipt and tumour location (for KRAS MT).

RFS analysis was performed using Cox proportional hazards method in the *BRAFMT, KRASMT* or *WT/WT* stratified by *Bcl-xL* expression levels. Analysis was performed both before and following adjustment. ^*^Cut-offs for low/medium/high *Bcl-xL* gene expression based on tertile values within each BRAF/KRAS status subgroup. ^**^Adjustments included age and sex, and were tested for TNM stage, MSI status, adjuvant chemotherapy receipt and tumor location for all models. A backwards elimination model was applied for tested confounders, until all were significant at the *p* < 0.25 level in the model. Final adjustments included age, sex, TNM stage and MSI status (for BRAF MT and WT/WT); age, sex, TNM stage, adjuvant chemotherapy receipt and tumor location (for KRAS MT).

In contrast, although there was a suggestive prognostic trend, no significant associations were observed for *Bcl-xL* gene expression in either the *KRASMT* or *WT/WT* patient groups, using either an adjusted or unadjusted analysis model (Table 3 and Supplementary Figure 3B and 3C).

### ZFAS1 mRNA expression is associated with poor prognosis in stage II/III BRAFMT CC

High gene expression of *ZFAS1* was associated with a prognostic trend in *BRAFMT* tumors compared to low gene expression (Supplementary Figure 4A) although given the small number of events in this stratified group, this trend failed to reach significance in either unadjusted HR = 4.69 (95% CI 0.52–42.01), or adjusted HR = 4.71 (95% CI 0.50–44.00) analyses (Supplementary Table 3). There was no prognostic value associated with high *ZFAS1* expression in the *KRASMT* population (adjusted HR = 0.65 (95% CI 0.34–1.24)), although there was a significant association with lower relapse rates in the WT/WT population (adjusted HR = 0.47 (95% CI 0.24–0.92)) (Supplementary Figure 4B, Supplementary Table 3) indicating an opposing prognostic role in these distinct tumor genotypes.

### Bcl-xL gene and protein expression are associated with the epithelial component of the tumor

**Figure 2:**
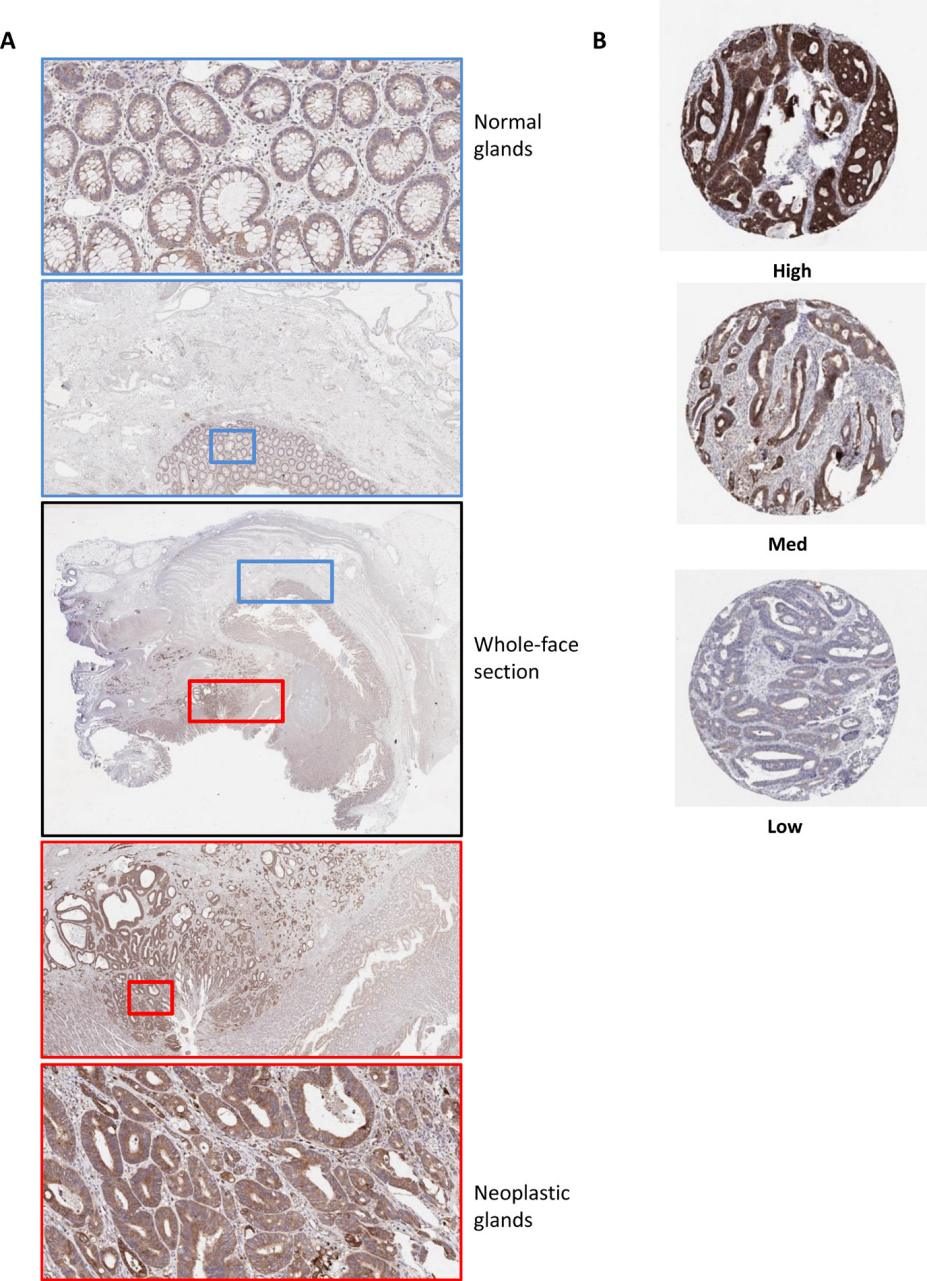
Optimization of immunohistochemistry staining protocol for Bcl-xL protein expression in CC (**A**) Whole-face CC tissue sections were used to optimize IHC protocol. A low level of protein expression was observed in the normal glands compared to surrounding stroma (Blue box) Elevated levels of expression were observed in neoplastic glands compared to both the normal glands and surrounding stroma (Red box). Some staining in the stroma is evident in both normal and cancer-associated regions. (**B**) Representative images of high, medium and low Bcl-xL protein expression by IHC in an independent “Northern Ireland cohort” of stage II/III CRC (Northern Ireland cohort; *n* = 740).

Given the multiple cell types that constitute the tumor microenvironment (TME) in CC, we analyzed *Bcl-xL* mRNA expression levels in transcriptional data derived from micro-dissected tumor tissue (detailed in Materials and Methods section). We observed that its expression was bimodal in the epithelial compartment of the TME, with high and low subgroups around the median, whereas stromal expression levels were generally low, with values below the median (Supplementary Figure 5A). Analysis of matched Bcl-xL transcript abundance (by Agilent microarray), and protein level, (by Reverse Phase Protein Array (RPPA)) from 102 CRC tumor samples within The Cancer Genome Atlas (TCGA) indicated a significant correlation between both methodologies (*p* = 0.001; Supplementary Figure 5B), supporting protein-based assessment as an appropriate methodology to validate our data in an independent cohort. Following optimization of an IHC protocol for *Bcl-xL* protein expression, the predominantly epithelial-derived nature of *Bcl-xL* protein expression and neoplastic-specific staining compared to the normal glands in surrounding tissue was confirmed in a series of whole-face CC sections, although there does appear to be some stromal expression, in line with our transcriptional analysis (Figure 2A).

### Independent validation of Bcl-xL as a poor prognostic marker specifically in stage II/III BRAFMT CC

We then independently validated the prognostic value of *Bcl-xL* protein expression specifically in *BRAFMT* patient samples from within a Northern Ireland cohort (*n* = 661) (Supplementary Figure 1 and described in Methods). Employing tertiles defined by protein expression (Figure 2B), we found that *Bcl-xL*^high^ was associated with an increased risk of CRC disease-specific survival (DSS; *n* = 77) when compared with *Bcl-xL*^low^, in both unadjusted (HR = 3.07 (95% CI 1.24–7.60)) and adjusted models (HR = 5.50 (95% CI 1.71–17.69) (Supplementary Figure 6A and Table 4). Similar findings were evident when using OS (*n* = 92) as the endpoint (Supplementary Figure 6B).

Table 4Analyses of disease-specific survival in the independent IHC validation cohort

**Table:** 

Bcl-xL	Alive *n*	CRC Death (DSS) *N*	Unadjusted Hazard ratios (95% confidence intervals)	Adjusted^**^ Hazard ratios (95% confidence intervals)
**BRAF MT**				
Low (<56.1)	16	7	1.00	1.00
Medium (56.1–<91.1)	17	10	1.54 (0.58–4.09)	1.97 (0.60–6.44)
High (≥91.1)	12	15	3.07 (1.24–7.60)	5.50 (1.71–17.69)
**KRAS MT**				
Low (<53.5)	44	28	1.00	1.00
Medium (53.5–<92.7)	41	24	0.98 (0.57–1.69)	0.93 (0.52–1.66)
High (≥92.7)	38	33	1.37 (0.82–2.27)	1.00 (0.57–1.77)
**WT/WT**				
Low (<66.1)	57	28	1.00	1.00
Medium (66.1–<105.8)	58	29	0.99 (0.59–1.68)	1.05 (0.60–1.84)
High (≥105.8)	59	31	1.07 (0.64–1.78)	1.18 (0.67–2.09)

MT: Mutant; WT/WT: BRAF and KRAS wild-type.

^*^Cut-offs for low/medium/high Bcl-xl gene expression based on tertile values within each BRAF/KRAS status subgroup.

^**^Adjustments included age, sex, TNM stage, MSI status, adjuvant chemotherapy receipt, ECOG status, family history of colorectal cancer, year of diagnosis and extramural venous invasion for all models.

**Table:** 

Bcl-xL	No Chemotherapy receipt			Chemotherapy receipt		
	Alive *n*	CRC Death (DSS) *N*	Adjusted Hazard ratios (95% confidence intervals)	Alive *n*	CRC Death *n*	Adjusted Hazard ratios (95% confidence intervals)
**BRAF MT**						
Low (<56.1)	11	4	1.00	5	3	1.00
Medium (56.1–<91.1)	12	6	1.99 (0.38–10.29)	5	4	2.18 (0.23–20.89)
High (≥91.1)	3	12	12.13 (2.49–59.13)	9	3	0.96 (0.08–11.42)
P for interaction			0.006			

MT: Mutant.

Cut-offs for low/medium/high Bcl-xl gene expression based on tertile values within each BRAF MT subgroup. Adjustments included age, sex, TNM stage, MSI status, adjuvant chemotherapy receipt, ECOG status, family history of colorectal cancer, year of diagnosis and extramural venous invasion.

(Top) DSS analysis was performed using Cox proportional hazards method in the *BRAFMT*, *KRASMT* or *WT/WT* stratified by Bcl-xL IHC (H-score) protein expression levels. Analysis was performed both before and following adjustment.

^*^Cut-offs for low/medium/high *Bcl-xL* gene expression based on tertile values within each BRAF/KRAS status subgroup.

^**^Adjustments included age, sex, TNM stage, MSI status, adjuvant chemotherapy receipt, ECOG status, family history of colorectal cancer, year of diagnosis and extramural venous invasion for all models. (Bottom) Further adjusted analysis to identify treatment interaction effect of the Bcl-xL-high tertile group of BRAFMT tumors stratified by treatment received.

**Figure 3:**
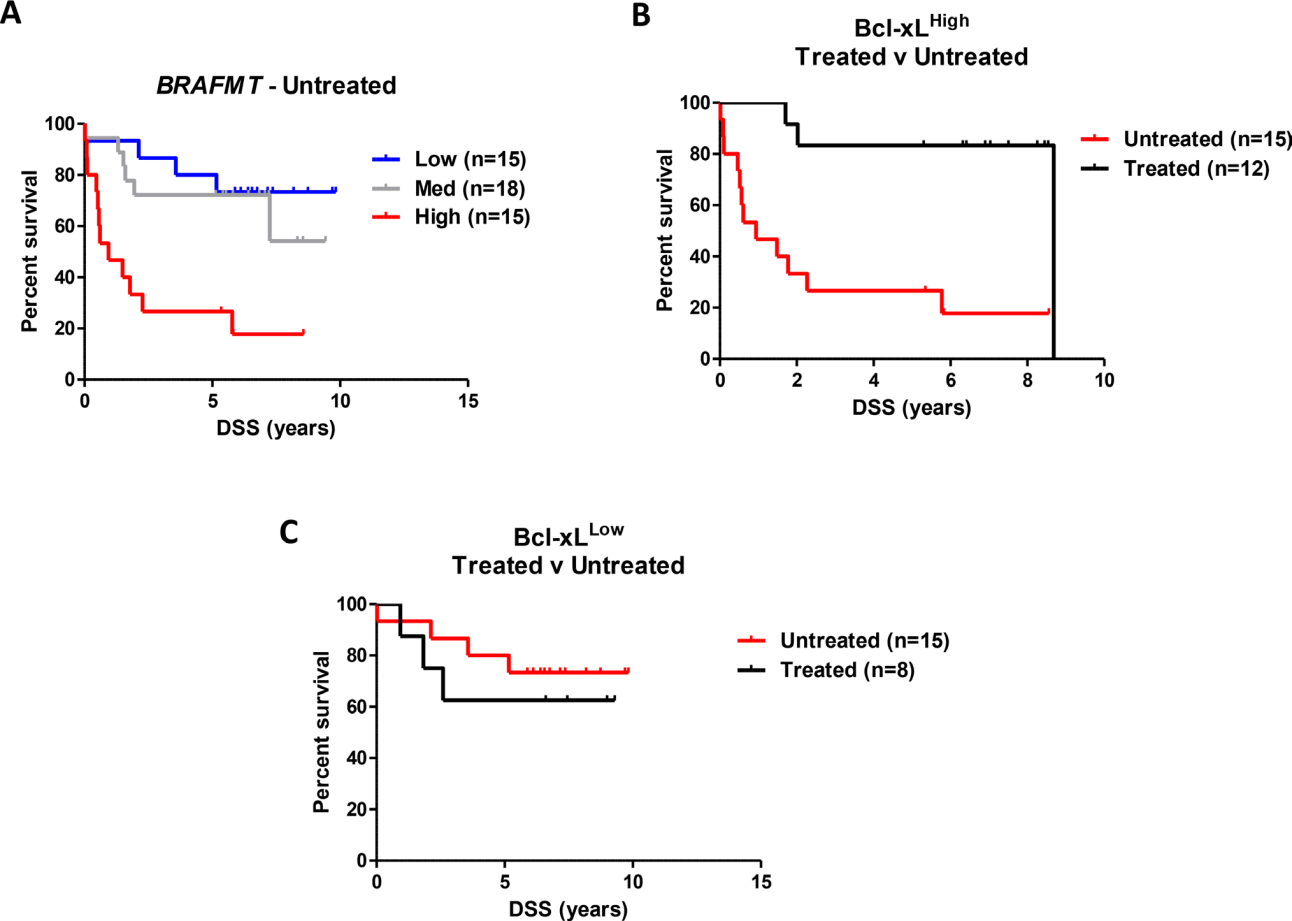
Independent validation of the prognostic value of Bcl-xL protein expression in *BRAFMT* CC (**A**) Colorectal cancer disease-specific survival (DSS) curve using Kaplan-Meier estimation in the “Northern Ireland cohort” comparing tertiles stratification of *Bcl-xL* protein expression (by IHC H-score) in untreated *BRAFMT* stage II/III CC patients. (**B**) DSS of patients in the highest tertile of *Bcl-xL* protein expression stratified according to adjuvant chemotherapy treatment received. (**C**) DSS of patients in the lowest tertile of *Bcl-xL* protein expression stratified according to adjuvant chemotherapy treatment received. Unadjusted and adjusted HR statistics are detailed in Table 4.

We next conducted stratified analyses within the Northern Ireland cohort to assess independently the prognostic value of Bcl-xL protein expression in both untreated and chemotherapy-treated *BRAFMT* patients. In untreated patients, we observed a 12-fold increased DSS risk in patients with the highest Bcl-xL protein expression (adjusted model HR = 12.13 (95% CI 2.49–59.13)) (Figure 3A), which was not observed in treated patients, (adjusted model HR = 0.96 (95% CI 0.08–11.42)) (Supplementary Figure 6C and Table 4). This significant prognostic benefit from adjuvant chemotherapy in *BRAFMT* patients was only observed in patients with the highest Bcl-xL protein expression (*P*-value for interaction = 0.006), whereas patients with low Bcl-xL protein expression derived no benefit from the addition of chemotherapy (Figure 3B and 3C, Table 4 and Supplementary Figure 6C). Similar results were evident when using OS as the endpoint (Supplementary Figure 6D–6F). Importantly, in agreement with our initial consolidation cohort, we were again able to confirm that the prognostic value of Bcl-xL protein expression was not observed in *KRASMT* (HR = 1.00 (95% CI 0.57–1.77) and *WT/WT* (HR = 1.18 (95% CI 0.67–2.09)) patient samples (Table 4).

## DISCUSSION

In this study, we set out to identify factors influencing patient prognosis specifically in tumors harboring an oncogenic *BRAF* mutation. Stratification of a discovery prognostic cohort based on risk-of-relapse identified the *Bcl-2* family member, *Bcl-xL*, as being upregulated at the transcriptional level in *BRAF*MT tumors from patients who went on to relapse following surgery, compared to those *BRAFMT* patients who experienced no disease recurrence. We validated the prognostic value of *Bcl-xL* specifically in *BRAFMT* tumors in both a consolidation transcriptional cohort and in an independent population-based stage II/III cohort. Importantly, in each validation series, we also confirmed the *BRAFMT*-specific nature of this association, as in either *KRASMT* or *WT/WT* tumors, the expression of *Bcl-xL* was not associated with increased risk of disease relapse or death. Interestingly, we observed that although *BRAFMT* tumors with high *Bcl-xL* expression have a poor prognosis, this subgroup also appears to benefit the most from standard adjuvant chemotherapy.

The prognostic value of stratifying CC patients based on *BRAF* mutational status has been well reported, particularly in stage IV metastatic tumors, where patients with *BRAFMT* tumors have poor survival rates. A previous study identified a transcriptional signature that could stratify stage II and III CRC tumor profiles into subgroups based on their similarity to *BRAFMT* tumors (pred-BRAFm) [7]. The authors demonstrated the utility of either *BRAF* mutational status or the pred-BRAFm classifier in identifying patients with shorter survival, although no difference was observed in the initial disease-specific relapse rates between the identified subgroups. This important result suggests that the prognostic power associated with the pred-BRAFm signature, or indeed the presence of the *BRAF* mutation itself, is due to shorter survival time because of aggressive disease *after* relapse in stage IV disease; however, initially, *BRAFMT* stage II and III patients are not at a higher risk of their early-stage disease progressing to metastatic disease. This subtle but crucial point underpins our rationale for performing a stratified analysis to identify factors determining risk-of-relapse specifically *within* the *BRAFTMT* genotype. The data presented here identifies for the first time a novel role for *Bcl-xL* expression in influencing disease relapse, providing a new, important and clinically relevant understanding of the biology underpinning aggressive *BRAFMT* stage II/III disease. Interestingly, we found almost no overlap between the genes associated with relapse in *BRAFMT* and *KRASMT* tumors, suggesting that although there is constitutive activation of the MAP kinase pathway in both these subgroups, there is clearly distinct prognostic tumor biology associated with these different genotypes.

The benefits of combining transcription-array discovery followed by IHC validation in independent patient cohorts, as we have employed in our study, was recently demonstrated in an analysis of stage II/III CC to identify a subgroup of undifferentiated tumors characterized by poor differentiation and low expression of the transcription factor *CDX2* [8]. There are a number of parallels between the *CDX2* study and our own, as they both use exploratory and retrospective analysis followed by clinically relevant IHC biomarker validation to identify a small subgroup of stage II/III patients with poor prognosis that appear to respond to adjuvant chemotherapy. Poorly differentiated tumors have previously been associated with right-sided MSI, CIMP disease [9]; however, the prognostic value of *BRAF* mutation was shown to be independent of *CDX2* expression [10]. Our analysis did not identify *CDX2* gene expression as a driver of disease relapse specifically in *BRAFMT* tumors and reciprocally *Bcl-xL* was not identified as prognostic across the entire CC population in the *CDX2* study (similar to our data in *BRAFWT* tumors), thus suggesting that we have identified a unique subgroup of poor prognostic patients. However, as the *CDX2* study did not collect or utilize *BRAF* mutation status, this could not be further assessed using their data.

A previous study of Bcl-xL protein expression in CRC determined that high expression of this biomarker was associated with poor prognosis across the entire patient cohort [11]. Importantly, this data indicated a potentially confounding variable, as increased Bcl-xL expression was also significantly associated with later stage disease (already a well-established prognostic factor) [12]. In agreement with this earlier study, we also found that Bcl-xL expression was associated with later stage disease; however, using an adjusted analysis to take into account known confounding clinical factors (including stage), we show that Bcl-xL expression can independently predict prognosis, but only in *BRAFMT* tumors. A recent study using RPPA methodology reported that a mathematical model of Bcl-2 family protein interactions (including Bcl-xL) termed DR_MOMP was prognostic in chemotherapy-treated stage III CRC [13]. Moreover, this study found that Bcl-2 family signaling was particularly important in Consensus Molecular Subtypes (CMS) 1 and 3. As the CMS1 subgroup is enriched for *BRAFMT* disease, this report appears to be in agreement with our current study. However, the individual contribution of Bcl-xL expression to prognosis in CMS1/*BRAFMT* CRC was not reported; the study may have been underpowered in that respect.

The reason for the significant benefit from standard chemotherapy of Bcl-xL high *BRAFMT* CRC is unclear. High Bcl-xL expressing tumors may be “primed” to undergo apoptosis in response to chemotherapy, due to co-expression of pro-apoptotic Bcl-2 family members. [14, 15] A recent high-throughput drug screen aimed at uncovering therapeutic strategies in CRC, revealed the essentiality of MCL1, Bcl-2 and Bcl-xL in *BRAFMT*-driven disease [16]. Additionally, a further drug screening-based study identified Bcl-xL as a critical regulator of MEK inhibitor resistance, which was synthetically lethal across a broad panel of *BRAFMT* cell line models [17]. These findings and the findings presented in our study suggest that directly targeting Bcl-xL may be an effective therapeutic strategy for *BRAFMT* CRC in the adjuvant disease setting.

We also identified high expression of the long noncoding RNA *ZFAS1* as a poor prognostic marker in our discovery dataset. *ZFAS1* was previously reported to be overexpressed in CRC compared to adjacent non-CRC tissue, with siRNA-mediated targeting revealing its role as a regulator of p53 protein levels, cell proliferation and colony formation in a small panel of CRC cell lines [18]. Validation of this marker, using methodologies such as RNA *in situ* hybridization, may clarify its role in disease progression and may become increasingly important as our understanding of the biology of long noncoding RNAs increases.

The findings presented both here and by others suggest that *BRAFMT* driven CRC is more aggressive than *BRAFWT* disease, but only when the disease has disseminated from the primary site. Interestingly, we observed specific changes in the ER-stress machinery in *BRAFMT* tumors with the highest-risk of disease relapse, with activation and upregulation of factors including GADD34, heat shock proteins, and stress-related transcription (DDIT3) in our analysis. Additionally, using GSEA, we identify increased hypoxia and EMT signaling in high-risk tumors, again indicating an association with ER stress-activation. Each of these factors have been demonstrated to activate the unfolded protein response (UPR), which in turn has been correlated with a higher risk of metastatic recurrence in breast cancer [19, 20]. In agreement with our findings, upregulation of UPR signaling in disseminated tumor cells from breast cancer, lung cancer and prostate cancer enables both the formation and long term persistence of metastatic lesions [19, 20]. In addition to activation of the UPR machinery, high Bcl-xL expression may promote survival of invasive tumor cells during the metastatic process; for example Bcl-xL has been reported to be a suppressor of anoikis, [15, 21] which would explain its association with increased risk-of-relapse in the *BRAFMT* subgroup.

This study has several strengths. We have identified Bcl-xL as a novel predictor of response within a poor prognostic group of CC patient samples, using a robust approach that included validation in an independent cohort using a clinically relevant methodology. Importantly, while we do find significant prognostic and predictive value using Bcl-xL gene expression in 2 independent cohorts, final validation of this discovery approach would require transcriptional data, detailed treatment information and clinical follow up from an independent well balanced cohort, preferably in a clinically trial setting, enriched for the specific subtype of interest, in our case *BRAFMT* stage II/III CRC. The population-based nature of our validation cohort also means that the results should be generalizable to all stage II/III CRC patients, however we do acknowledge that by using a population-based approach for validation of these findings, there may be a selection bias for patients who subsequently received chemotherapy, and this that may have impacted on our results. Additionally, given that IHC and mutational tests for *BRAF* and *KRAS* are routinely utilized in the diagnostic work-up of CC patients, the methods we have used here could easily be employed within routine pathology reporting practice. However, we do acknowledge that further work is required to identify an optimal cut-off level of *Bcl-xL* expression that would allow a more robust classification of low and high expressers for prospective patient stratification.

In conclusion, we have identified and independently validated the prognostic value of Bcl-xL mRNA and protein expression specifically within *BRAFMT* CC, which should help inform selection of treatment options for high-risk *BRAFMT* stage II/III patients in the adjuvant disease setting. This approach could prevent the initial relapses, which ultimately contribute to the poor outcomes of patients with this genotype. Data presented here provide compelling evidence that, in addition to *BRAF* mutational analysis, assessment of Bcl-xL protein expression using routine diagnostic IHC methods can identify both poor prognostic *BRAFMT* stage II/III CC patients who will benefit from adjuvant therapy and an otherwise good prognostic subgroup of *BRAFMT* patients who derive no significant advantage from the addition of adjuvant chemotherapy.

## MATERIALS AND METHODS

### Transcriptional datasets

Gene expression profiles were downloaded from NCBI Gene Expression Omnibus (GEO) (http://www.ncbi.nlm.nih.gov/geo/). Accession number GSE39582 contains 566 tumor transcriptional profiles (460 stage II/III) from a CC series and has previously been employed by the CRC subtyping consortium [22, 23]. As detailed in Supplementary Figure 1, the GSE39582 cohort contained 460 stage II/III CC profiles which had relapse data available. For initial biomarker discovery, the “Prognostic Subset” contained untreated stage II/III patients stratified into high-risk (if the patient relapsed within 36 months) or low-risk (if there was no relapse). The “Initial Consolidation” contained all stage II/III patients with relapse information and mutational data (*n* = 417), which included *BRAFMT* (*n* = 41; 24 of which will have been used already in the prognostic subset), *KRASMT* (*n* = 166) or *WT/WT* (*n* = 210) (Supplementary Figure 1). Accession number GSE35602 contains profiles from 13 CRC cases, which were obtained using laser-microdissected tissue to extract RNA specifically from stroma or epithelium regions separately, followed by gene expression microarray analysis.

Transcriptomics (Agilent; mRNA_Preprocess_Median) and protein (Reverse Phase Protein Array/mda_rppa_core-protein_normalization) data were downloaded from the COAD pipeline in Firehose (https://gdac.broadinstitute.org/). Patient samples which had both mRNA and RPPA data were collated (*n* = 102) and were analysed with the Pearson’s correlative analysis using GraphPad Prism version 5 for Windows.

### Transcriptional analysis

Partek Genomics Suite was employed for dataset analysis. Differentially expressed probesets which had a fold-change +/– 1.75 fold and *p*-value < 0.005 were defined using analysis of variance (ANOVA) of supervised risk groupings in both the *BRAFMT* and *KRASMT* subgroups separately. Genes represented three times by different probesets were selected for further genotype-specific survival analysis. This method inevitably increases false negatives, by ruling out genes represented by fewer probesets, but it increases the confidence in the positive results. In the *BRAFMT* analysis, these criteria identified *BCL2L1* (*Bcl-xL*) and *NCRNA00275* (*ZFAS1*). Gene Set Enrichment Analysis was accessed (GSEA; http://software.broadinstitute.org/gsea/index.jsp) and the Microenvironment Cell Populations-counter (MCP) was accessed via the https://doi.org/10.5281/zenodo.61372 link.

### Bcl-xL Immunohistochemistry (Bcl-xL IHC)

We optimized a protocol for Bcl-xL IHC on sections of CRC tissue using various antibody dilutions and processing parameters. In line with REMARK guidelines, reproducibility and robustness were tested using a TMA block containing 20 cores of CRC tumor from different patient resections. For staining of the control and independent cohort TMAs, sections were cut at 4 μm on a rotary microtome, dried at 37° C overnight, and then used for IHC, which was performed on an automated immunostainer (Leica Bond-Max, Milton Keynes, UK). Antigen-binding sites were detected with a polymer-based detection system (Bond, Newcastle Upon Tyne, UK; cat. no. DS9800). *Bcl-xL* IHC antibody (Cell Signaling Technology, MA, United States) (*Bcl-xL* (54H6) Rabbit mAb #2764) was employed at 1:250 dilution with epitope retrieval solution 2 pretreatment for 30 minutes. All sections were visualized with diaminobenzidine, counterstained with hematoxylin, and mounted in DPX.

### Independent stage II/III CC Northern Ireland validation cohort

Candidate biomarkers identified from transcriptional datasets were then evaluated within a Northern Ireland population-based cohort of stage II/III CC patient samples (*n* = 740) using immunohistochemical methods. The Northern Ireland Cancer Registry was used to identify all patients who underwent surgery in Northern Ireland between 2004 and 2008, for a single, primary, stage II or III colon adenocarcinoma (*n* = 1,539). A detailed clinical case note review was then conducted, to verify diagnosis and stage and to extract clinical information, including the use of adjuvant chemotherapy and outcome data. Following this review, *n* = 113 cases were excluded (7%), mainly on the basis of inaccurate staging. Of the remaining *n* = 1,426 patients, *n* = 740 patients (52%) were diagnosed within the jurisdiction of the Northern Ireland Biobank, of which specimens relating to *n* = 661 patients (89%) were successfully retrieved. All patients were followed up for occurrence and cause of death via linkage to the Northern Ireland Registrar General’s Office, up to 31st December 2013. Patients were recorded as having a CRC-specific death if any cause of death was listed as ICD-codes C18, C19, C20 and/or C26.

### Northern Ireland cohort immunohistochemical and mutational analysis

This cohort was assembled into a tissue microarray, containing 3 cores from epithelial-rich tumor regions per patient. Blocks were retrieved and tumor regions were annotated for subsequent coring (KA, MBL, JJ). One millimeter diameter tissue cores were extracted from donor blocks and inserted into recipient blocks using a manual tissue arrayer (Estigen, Tartu, Estonia). Additionally, mutational analysis was undertaken for KRAS and BRAF on *n* = 661 (89%) of the TMA cohort using the ColoCarta panel (Agena Bioscience, Hamburg, Germany). This panel includes: BRAF: D594V, V600E, V600K, V600L, V600R. HRAS Q61L. KRAS: A59T, G12A, G12C, G12D, G12F, G12R, G12S, G12V, G13D, G61H, Q61L. Following sequencing, mutational status of *BRAF* and *KRAS* was available for a sub-cohort (*n* = 661; *BRAFMT n* = 92, *KRASMT n* = 248, *WT/WT n* = 321). Using the IHC methodology optimized in line with the REMARK guidelines in whole face CC sections, we assessed *Bcl-xL* protein expression using digital pathology software, QuPath [24], to give a numerical representation of both the extent and the intensity of staining (H-score), based on the mean expression of all cores (3 cores for each patient). In line with REMARK guidelines, all scoring was performed while blinded to the clinical details of the cohort and the survival endpoints. Using tertile stratification methodology, we assigned patients in each mutational genotype into high, medium or low groups according to their Bcl-xL protein expression H-score.

### Ethical approval

Clinical note review was conducted under the auspices of the Northern Ireland Cancer Registry ethical approval from ORECNI (REC: 10/NIR02/53). Ethical (REC:11/NI/0013, project NIB13-0069) and Bcl-xL staining (NIB16-0212) approval was received from the Northern Ireland Biobank.

### Statistical analysis

Clinical characteristics were compared using chi-squared tests, according to mutational groupings. Tertile stratification in GSE39582 was performed on the mean biomarker, BCL2L1 (*Bcl-xL*), expression value from the three probesets used within the *BRAFMT* (*n* = 42), *KRASMT* (*n* = 166) and the *WT/WT* (*n* = 210) subgroups. Similarly, in the Northern Ireland cohort, tertile stratification was performed on the mean *Bcl-xL* H-score expression value from the multiple tumor cores available (up to 3 per patient) within the *BRAFMT* (*n* = 92), *KRASMT* (*n* = 248) and the *WT/WT* (*n* = 321) subgroups. Cox Proportional hazards analysis was conducted for both the transcriptional dataset and Northern Ireland cohort, prior to and after adjustment for potential confounders, to evaluate the association between *Bcl-xL* and survival in CC patients, according to *BRAF* and *KRAS* mutation status (Stata version 11.2, StataCorp, College Station, TX, USA).

## SUPPLEMENTARY MATERIALS FIGURES AND TABLES






