# Supplementary material for: Bcl-xL as a poor prognostic biomarker and predictor of response to adjuvant chemotherapy specifically in BRAF-mutant stage II and III colon cancer
Source: Oncotarget. 2018 Feb 13;9(17):13834–47. doi: 10.18632/oncotarget.24481 (PMC5862619; doi:10.18632/oncotarget.24481)
Supplement: Supplementary file 1 [file oncotarget-09-13834-s001.pdf]

Bcl-xL as a poor prognostic biomarker and predictor of response to adjuvant chemotherapy specifically in *BRAF*-mutant stage II and III colon cancer

SUPPLEMENTARY MATERIALS

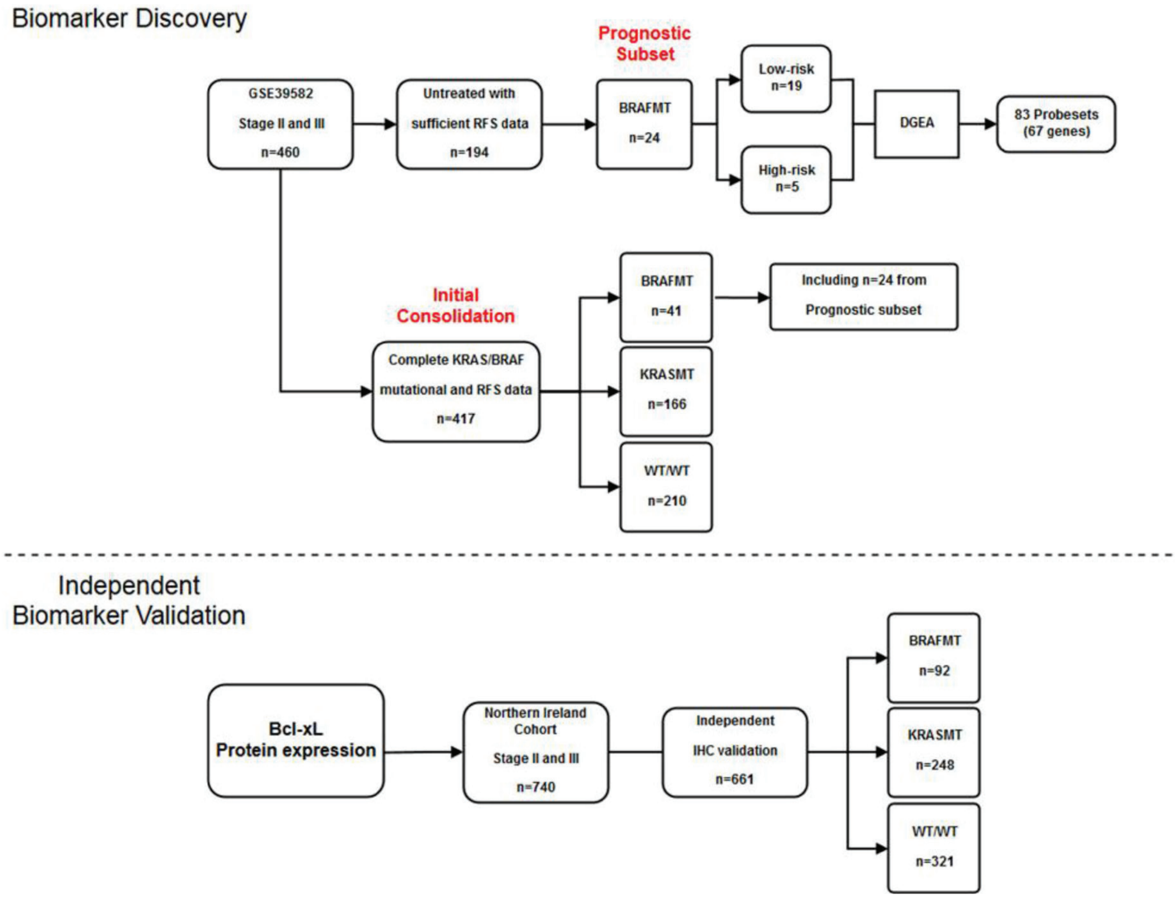

Supplementary Figure 1: Study overview of discovery and survival validation subsets. Biomarker Discovery: The data from GSE39582 was accessed through the NCBI GEO portal (https://www.ncbi.nlm.nih.gov/geo/). From the 566 Affymetrix U133 Plus 2.0 patient transcriptional profiles within this accession number, we selected profiles from stage II/III tumors with complete relapse data ($n = 460$ profiles). The "Prognostic Subset" was composed of *BRAFMT* and *KRASMT* tumors, which fulfilled risk filtering (see Methods) followed by differential gene expression analysis based on risk classification. *Bcl-xL* (and *ZFAS1*) were selected for relapse-free survival analyses using all stage II/III patients to create an "Initial Consolidation" subset ($n = 417$), which was composed of either *BRAFMT* ($n = 41$), *KRASMT* ($n = 166$) or *WT/WT* ($n = 210$) subgroups of samples. Independent Biomarker Validation: Patients from the Northern Ireland stage II/III cohort ($n = 740$) with clinical follow up and mutational status ($n = 661$) formed the "Independent IHC validation" cohort (described in detail in Methods) and contained *BRAFMT* ($n = 92$), *KRASMT* ($n = 248$) and *WT/WT* ($n = 321$) subgroups of patients.

A

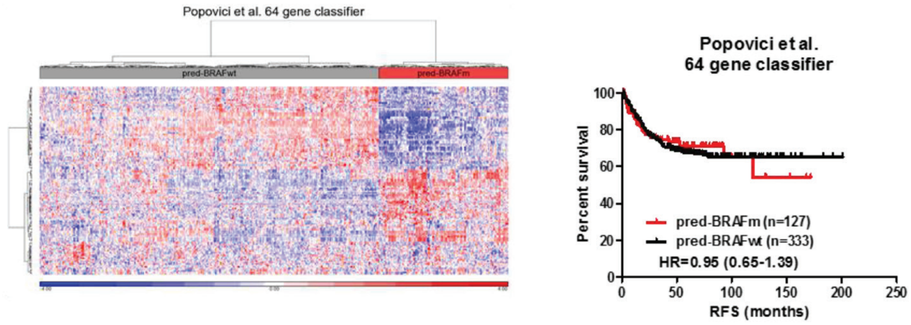

B

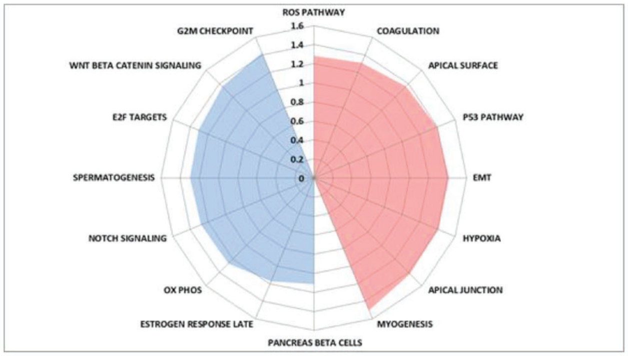

C

Cell Type	p-value(High vs. Low)	Ratio(High vs. Low)	Fold-Change(High vs. Low)	95% lower limit	95% upper limit
Fibroblasts	0.07	1.62	High up vs Low	-1.04	2.73
Cytotoxic lymphocytes	0.33	1.20	High up vs Low	-1.21	1.74
Monocytic lineage	0.36	1.20	High up vs Low	-1.24	1.79
Neutrophils	0.52	0.93	High down vs Low	-1.36	1.17
B lineage	0.54	0.85	High down vs Low	-2.06	1.48
NK cells	0.58	0.96	High down vs Low	-1.21	1.11
Endothelial cells	0.66	1.04	High up vs Low	-1.15	1.24
Myeloid dendritic cells	0.74	0.97	High down vs Low	-1.28	1.19
CD8 T cells	0.76	0.95	High down vs Low	-1.53	1.37
T cells	0.80	1.03	High up vs Low	-1.26	1.34

D

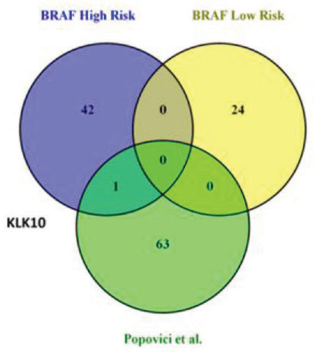

E

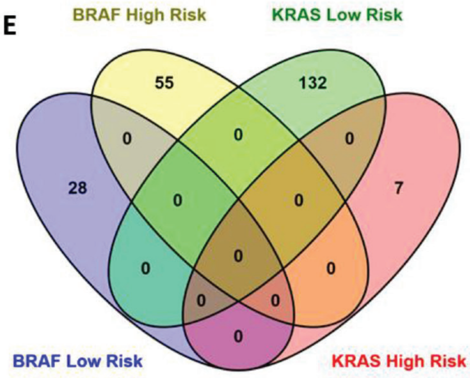

Supplementary Figure 2: Relapse risk analysis of previously published *BRAF* signature. (**A**) (Left) Hierarchical clustering using Ward and Euclidean metrics based on gene expression profiles of the 64 gene signature[4] (pred-BRAFm) using stage II/III CC transcription profiles from GSE39582 (n = 460) identified 2 distinct subgroups. (Right) Kaplan-Meier relapse survival analysis of these 2 subgroups (n = 333 vs n = 127) revealed no significant difference in relapse rates. Hazard Ratio (HR) and confidence interval calculated using log-rank method. (**B**) Radar plot depicting the enrichment scores from GSEA (http://software.broadinstitute.org/gsea/index.jsp) of high-risk and low-risk tumors used in the prognostic subset analysis. (**C**) Microenvironment Cell Populations-counter (MCP; https://doi.org/10.5281/zenodo.61372) analysis of high-risk and low-risk tumors used in the prognostic subset analysis. (**D**) Venn diagram analysis of our *BRAFMT* relapse risk genes compared to the previously published pred-BRAFm 64-gene signature from Popovici et al. [4] (**E**) Venn diagram comparative analysis of genes associated with risk of relapses in either *BRAFMT* or *KRASMT* genotypes revealed no overlap in the prognostic biology of these genotypes.

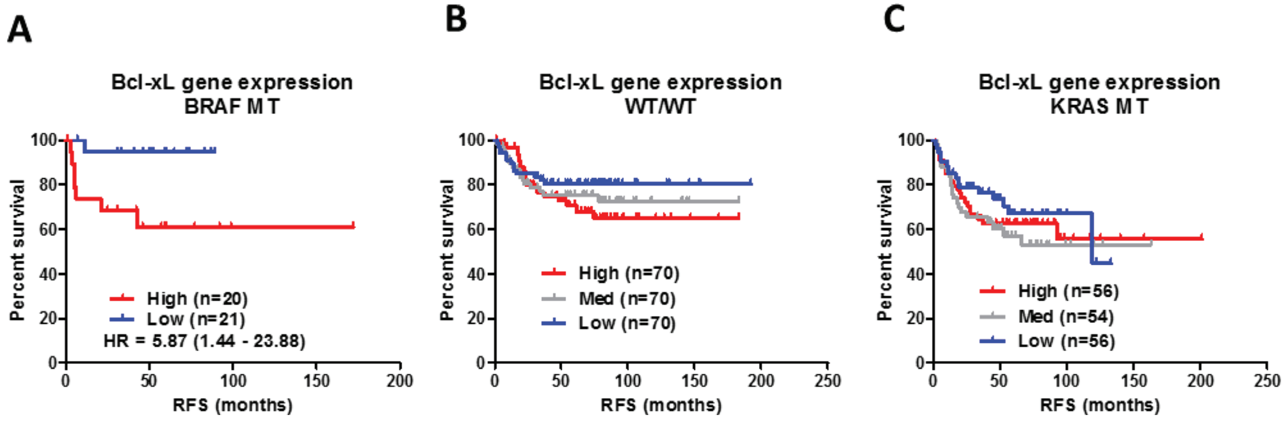

Supplementary Figure 3: Kaplan-Meier analyses of *Bcl-xL* relapse-free survival. (**A**) RFS analysis of *Bcl-xL* gene expression levels in *BRAFMT* tumors stratified on median gene expression. (**B**) RFS analysis of *Bcl-xL* gene expression levels in all *WT/WT* tumors. (**C**) RFS analysis of *Bcl-xL* gene expression levels in all *KRASMT* tumors. Unadjusted and adjusted HR statistics are detailed in Table 3.

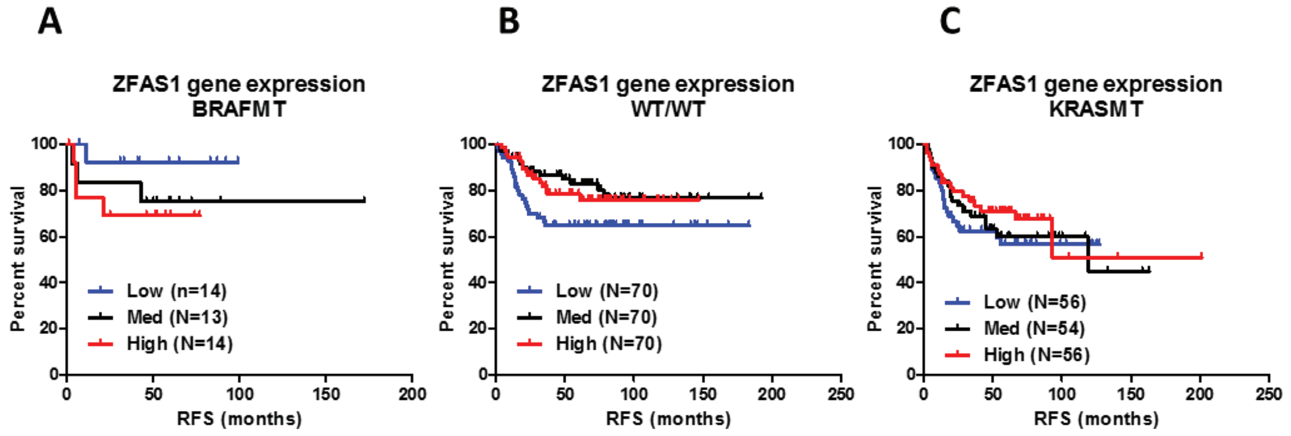

Supplementary Figure 4: Kaplan-Meier analyses of *ZFAS1* relapse-free survival. (**A–C**) RFS analysis of tertile stratified *ZFAS1* gene expression levels in *BRAFMT* (A), *WT/WT* (B) and *KRASMT* (C) stage II/III CRC patients (GSE39582). Unadjusted and adjusted HR statistics are detailed in Supplementary Table 3.

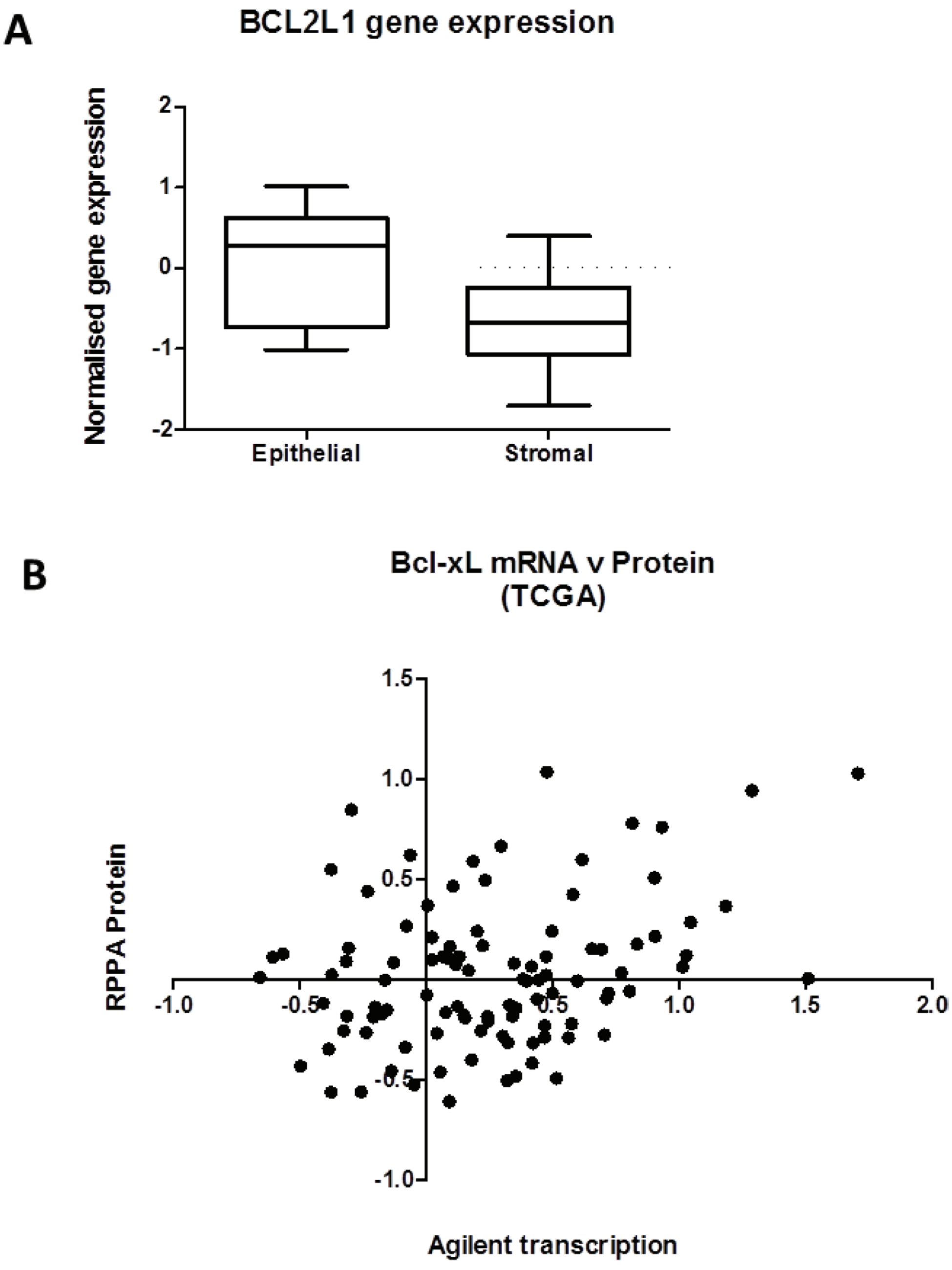

Number of XY Pairs	102
Pearson r	0.3129
95% confidence interval	0.1260 to 0.4783
P value (two-tailed)	0.0014
P value summary	**
Is the correlation significant? (alpha=0.05)	Yes

Supplementary Figure 5: *Bcl-xL* gene expression within the tumor microenvironment. (**A**) Analysis of *Bcl-xL* gene expression levels in stromal and epithelial components (GSE35602) highlights bimodal expression within the epithelial compartment. (**B**) Analysis of preprocessed mRNA (Agilent) and normalized protein (RPPA) data from the TCGA Firehose (https://gdac.broadinstitute.org/) indicated a significant correlation (Pearson's similarity) between *Bcl-xL* gene expression levels and protein expression.

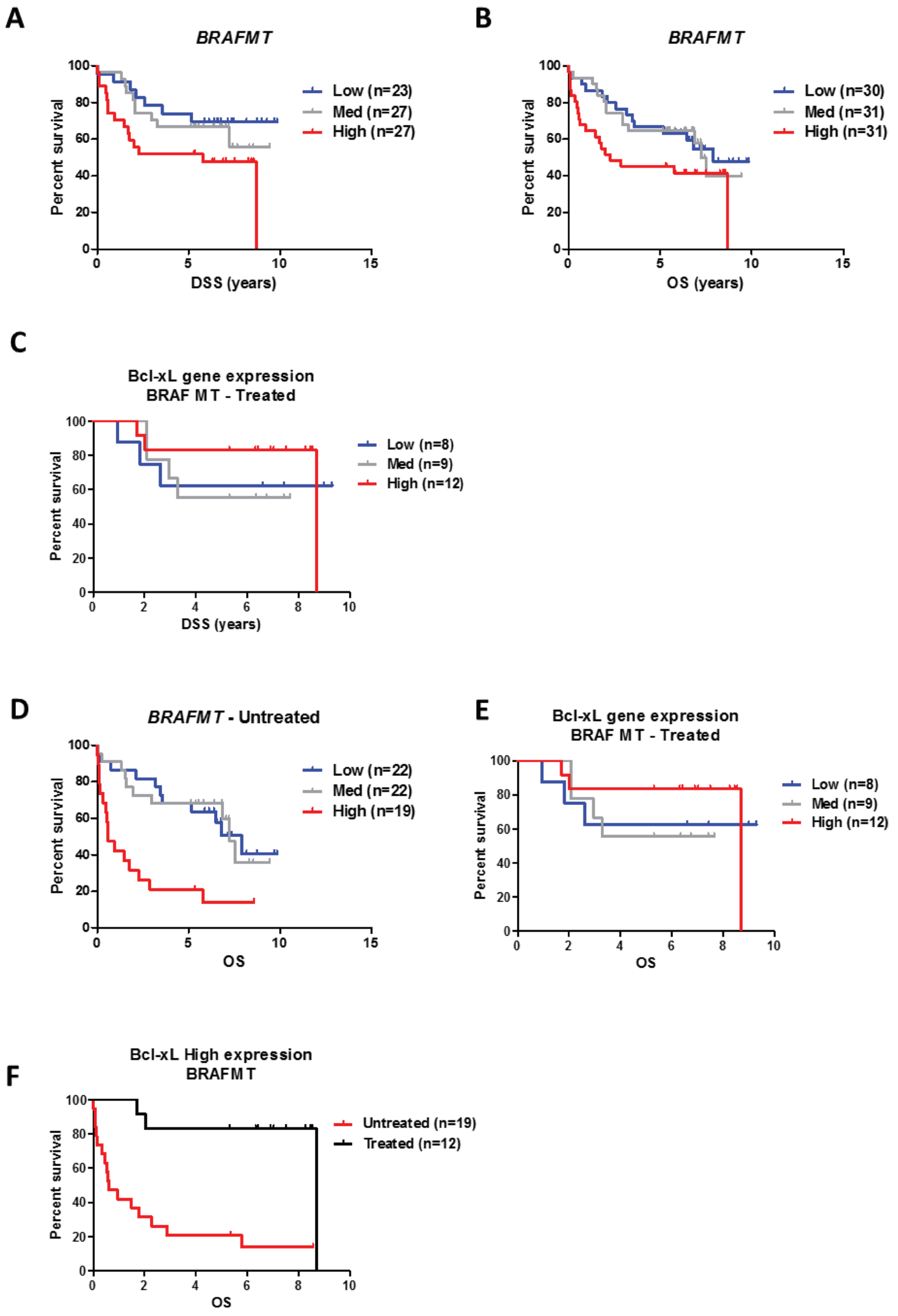

Supplementary Figure 6: Kaplan-Meier analyses of Bcl-xL overall- and disease-specific survival in independent validation cohort. (**A** and **B**) Overall survival (OS) and Disease-specific survival (DSS) curves using Kaplan-Meier estimation comparing tertile stratification of Bcl-xL protein expression (by IHC H-score) in all *BRAFMT* stage II/III CC patients. (**C**) DSS of Bcl-xL levels in treated *BRAFMT* tumors stratified by tertile. (**D** and **E**) Overall survival analysis of Bcl-xL protein expression levels in untreated and treated *BRAFMT* tumors stratified by tertile. Bcl-xL (**F**) Overall survival analysis of Bcl-xL-high protein expression in treated *BRAFMT* tumors. Unadjusted and adjusted HR statistics are detailed in Table 4.

Supplementary Table 1: Probesets significantly associated with relapse risk in *BRAFMT* tumors. See Supplementary_Table_1

Supplementary Table 2: Probesets significantly associated with relapse risk in *KRASMT* tumors. See Supplementary_Table_2

Supplementary Table 3: Unadjusted and adjusted analyses of relapse-free survival

ZFAS1	Non-progressors *n*	Progressors *n*	Unadjusted Hazard ratios (95% confidence intervals)	Adjusted** Hazard ratios (95% confidence intervals)
**BRAF MT**				
**Low**	13	1	1.00	1.00
**Medium**	10	3	3.50 (0.36–33.73)	3.09 (0.31–30.56)
**High**	10	4	4.69 (0.52–42.01)	4.71 (0.50–44.00)
**KRAS MT**				
**Low**	35	22	1.00	1.00
**Medium**	32	21	0.87 (0.47–1.62)	0.76 (0.41–1.42)
**High**	38	18	0.71 (0.37–1.33)	0.65 (0.34–1.24)
**WT/WT**				
**Low**	48	22	1.00	1.00
**Medium**	57	13	0.48 (0.24–0.95)	0.41 (0.20–0.82)
**High**	55	15	0.57 (0.30–1.10)	0.47 (0.24–0.92)

MT: Mutant; WT/WT: BRAF and KRAS wild-type.

*Cut-offs for low/medium/high NCRNA (ZFAS1) gene expression based on tertile values within each BRAF/KRAS status subgroup.

**Adjustments included age and sex, and were tested for TNM stage, MSI status, adjuvant chemotherapy receipt and tumour location for all models. A backwards elimination model was applied for tested confounders until all were significant at the $p < 0.25$ level in the model. Final adjustments included age, sex, and TNM stage (for BRAF MT); age, sex, TNM stage, adjuvant chemotherapy and tumour location (for KRAS MT); age, sex, TNM stage, MSI status and tumour location (for WT/WT).

RFS analysis was performed using Cox proportional hazards method in the *BRAFMT*, *KRASMT* or *WT/WT* stratified by *ZFAS1* expression levels. *Cut-offs for low/medium/high *ZFAS1* gene expression based on tertile values within each BRAF/KRAS status subgroup. **Adjustments included age and sex, and were tested for TNM stage, MSI status, adjuvant chemotherapy receipt and tumor location for all models. A backwards elimination model was applied for tested confounders until all were significant at the $p < 0.25$ level in the model. Final adjustments included age, sex, and TNM stage (for BRAF MT); age, sex, TNM stage, adjuvant chemotherapy and tumor location (for KRAS MT); age, sex, TNM stage, MSI status and tumor location (for WT/WT).
