# Supplementary material for: Bcl-xL as a poor prognostic biomarker and predictor of response to adjuvant chemotherapy specifically in BRAF-mutant stage II and III colon cancer
Source: Oncotarget. 2018 Feb 13;9(17):13834–47. doi: 10.18632/oncotarget.24481 (PMC5862619; doi:10.18632/oncotarget.24481)
Supplement: Supplementary file 2 [file oncotarget-09-13834-s002.docx]

**Supplementary Table 1:** **Probesets significantly associated with relapse risk in *BRAFMT* tumors**

| **Column ID** | **Gene Symbol** | **p-value(Risk)** | **Fold-Change(High vs. Low)** |
| --- | --- | --- | --- |
| 201792_at | AEBP1 | 0.00357813 | 2.39615 |
| 204664_at | ALPP | 0.0036019 | 1.88676 |
| 224339_s_at | ANGPTL1 | 0.000141463 | 1.92782 |
| 212312_at | BCL2L1 | 0.00253944 | 1.75675 |
| 206665_s_at | BCL2L1 | 0.00403036 | 1.841 |
| 215037_s_at | BCL2L1 | 0.00191145 | 1.9688 |
| 1569144_a_at | C9orf169 | 0.00268459 | 2.01757 |
| 208075_s_at | CCL7 | 0.000118381 | 2.22544 |
| 205627_at | CDA | 0.000109302 | 7.0876 |
| 209383_at | DDIT3 | 0.000454073 | 1.81158 |
| 200666_s_at | DNAJB1 | 0.00158798 | 1.8228 |
| 200664_s_at | DNAJB1 | 0.00021294 | 2.00779 |
| 224825_at | DNTTIP1 | 0.000217611 | 2.0873 |
| 234942_s_at | DNTTIP1 | 6.34E-05 | 2.1421 |
| 203367_at | DUSP14 | 0.00352545 | 1.95014 |
| 206439_at | EPYC | 0.00136055 | 3.66815 |
| 229521_at | FLJ36031 | 0.00446205 | 1.87711 |
| 226847_at | FST | 0.00409711 | 1.82151 |
| 224252_s_at | FXYD5 | 0.00452331 | 1.95374 |
| 204457_s_at | GAS1 | 0.00206559 | 4.65337 |
| 215243_s_at | GJB3 | 0.00398615 | 2.60134 |
| 206156_at | GJB5 | 0.00102843 | 2.39729 |
| 45714_at | HCFC1R1 | 0.00279056 | 1.82102 |
| 117_at | HSPA6 | 0.000228093 | 2.59902 |
| 213418_at | HSPA6 | 0.000539337 | 3.51734 |
| 226559_at | IER5L | 0.00289594 | 1.86633 |
| 203851_at | IGFBP6 | 0.0049927 | 3.44117 |
| 215808_at | KLK10 | 0.00157391 | 2.12964 |
| 209800_at | KRT16 | 0.00132379 | 2.30971 |
| 214580_x_at | KRT6A /// KRT6B /// KRT6C | 0.00213891 | 2.01611 |
| 244740_at | LOC100128252 | 0.000395352 | 1.78227 |
| 225381_at | LOC399959 | 0.00321655 | 5.10276 |
| 210605_s_at | MFGE8 | 0.00168213 | 1.93601 |
| 208148_at | MYH4 | 0.00187218 | 1.88317 |
| 226227_x_at | NCRNA00275 | 0.00102301 | 1.82659 |
| 224915_x_at | NCRNA00275 | 0.000814649 | 1.85619 |
| 226835_s_at | NCRNA00275 | 0.00101009 | 1.89756 |
| 225930_at | NKIRAS1 | 0.00158521 | 1.75339 |
| 220106_at | NPC1L1 | 0.00210857 | 1.9331 |
| 227486_at | NT5E | 0.000965098 | 2.69796 |
| 206859_s_at | PAEP | 0.00310726 | 3.14511 |
| 218273_s_at | PDP1 | 0.00474931 | 2.04182 |
| 222572_at | PDP1 | 0.00161747 | 2.24056 |
| 218634_at | PHLDA3 | 0.00337563 | 2.13164 |
| 202014_at | PPP1R15A | 0.00414088 | 1.7996 |
| 37028_at | PPP1R15A | 0.00489879 | 1.81077 |
| 237732_at | PRR9 | 0.00117195 | 1.8945 |
| 205228_at | RBMS2 | 0.001113 | 1.76487 |
| 204337_at | RGS4 | 0.00161388 | 3.08886 |
| 204743_at | TAGLN3 | 0.00295239 | 1.80623 |
| 228121_at | TGFB2 | 0.00279341 | 1.91989 |
| 207426_s_at | TNFSF4 | 0.00335822 | 2.3434 |
| 203683_s_at | VEGFB | 0.001539 | 1.77802 |
|  |  |  |  |
| **Column ID** | **Gene Symbol** | **p-value(Risk)** | **Fold-Change(High vs. Low)** |
| 209173_at | AGR2 | 0.00263761 | -2.7237 |
| 213143_at | C2orf72 | 0.00232002 | -1.98416 |
| 242447_at | C3orf70 | 0.000112081 | -3.83449 |
| 235562_at | C3orf70 | 0.00349908 | -1.95876 |
| 213050_at | COBL | 0.00351991 | -1.82387 |
| 214106_s_at | GMDS | 0.0045034 | -2.53458 |
| 226446_at | HES6 | 0.00336194 | -2.35981 |
| 203126_at | IMPA2 | 0.00231815 | -1.95056 |
| 226248_s_at | KIAA1324 | 0.0021877 | -6.44242 |
| 225525_at | KIAA1671 | 0.00233745 | -1.78939 |
| 227250_at | KREMEN1 | 0.00109032 | -1.75114 |
| 215543_s_at | LARGE | 0.000940792 | -1.97306 |
| 236118_at | LOC100128893 | 0.00240066 | -2.3928 |
| 238956_at | LOC100506781 | 0.000595672 | -3.19352 |
| 206418_at | NOX1 | 0.0036011 | -5.46402 |
| 207217_s_at | NOX1 | 0.00473879 | -3.70583 |
| 226499_at | NRARP | 0.00325207 | -2.25952 |
| 205251_at | PER2 | 0.00356648 | -2.22784 |
| 205632_s_at | PIP5K1B | 0.0020144 | -4.12775 |
| 242055_at | PSMG4 | 0.00289168 | -2.4234 |
| 203625_x_at | SKP2 | 0.0043362 | -1.75644 |
| 223194_s_at | SLC22A23 | 0.00277791 | -2.35077 |
| 212458_at | SPRED2 | 0.00151256 | -1.86388 |
| 213285_at | TMEM30B | 0.0019072 | -2.48282 |
| 36742_at | TRIM15 | 0.00348685 | -1.95849 |
| 217979_at | TSPAN13 | 0.0010721 | -2.12954 |
