# Supplementary material for: Bcl-xL as a poor prognostic biomarker and predictor of response to adjuvant chemotherapy specifically in BRAF-mutant stage II and III colon cancer
Source: Oncotarget. 2018 Feb 13;9(17):13834–47. doi: 10.18632/oncotarget.24481 (PMC5862619; doi:10.18632/oncotarget.24481)
Supplement: Supplementary file 3 [file oncotarget-09-13834-s003.docx]

**Supplementary Table 2: Probesets significantly associated with relapse risk in *KRASMT* tumors.**

| **Column ID** | **Gene Symbol** | **p-value(Risk)** | **Fold-Change(High vs. Low)** |
| --- | --- | --- | --- |
| 206208_at | CA4 | 0.00389026 | 1.81044 |
| 206209_s_at | CA4 | 0.00461504 | 2.39118 |
| 231814_at | MUC12 | 0.00247551 | 2.5351 |
| 231941_s_at | MUC20 | 0.00435644 | 1.91621 |
| 201481_s_at | PYGB | 0.00213036 | 1.85362 |
| 205464_at | SCNN1B | 0.000441448 | 1.84519 |
| 218345_at | TMEM176A | 0.000936925 | 1.85153 |
|  |  |  |  |
| **Column ID** | **Gene Symbol** | **p-value(Risk)** | **Fold-Change(High vs. Low)** |
| 228241_at | AGR3 | 0.00346503 | -2.75387 |
| 222108_at | AMIGO2 | 0.00322124 | -1.87098 |
| 201012_at | ANXA1 | 0.000205205 | -2.05865 |
| 204205_at | APOBEC3G | 8.38E-05 | -1.85521 |
| 235333_at | B4GALT6 | 1.30E-05 | -1.75234 |
| 209406_at | BAG2 | 0.00183076 | -2.08003 |
| 203685_at | BCL2 | 4.53E-05 | -1.79155 |
| 205681_at | BCL2A1 | 0.000757676 | -2.15802 |
| 210538_s_at | BIRC3 | 9.95E-06 | -2.12007 |
| 221478_at | BNIP3L | 2.30E-05 | -1.95538 |
| 238794_at | C10orf78 | 0.000127179 | -1.83311 |
| 1552701_a_at | CARD16 | 0.000128506 | -1.79847 |
| 1552703_s_at | CARD16 /// CASP1 | 0.000614458 | -1.83203 |
| 211368_s_at | CASP1 | 0.00146134 | -1.94838 |
| 206011_at | CASP1 | 0.00333069 | -1.93386 |
| 52285_f_at | CEP76 | 1.19E-06 | -2.11923 |
| 219311_at | CEP76 | 8.38E-07 | -1.83853 |
| 1555564_a_at | CFI | 0.000432979 | -2.36342 |
| 203854_at | CFI | 0.000791105 | -2.06731 |
| 235117_at | CHAC2 | 6.64E-06 | -2.01533 |
| 209732_at | CLEC2B | 0.000106237 | -1.82715 |
| 205159_at | CSF2RB | 0.000709512 | -1.76088 |
| 204533_at | CXCL10 | 0.000180474 | -2.52959 |
| 211122_s_at | CXCL11 | 0.00127775 | -3.03047 |
| 210163_at | CXCL11 | 0.000889939 | -2.72659 |
| 205242_at | CXCL13 | 0.00140635 | -2.44987 |
| 203915_at | CXCL9 | 0.000268626 | -2.56936 |
| 209606_at | CYTIP | 0.00113889 | -1.75922 |
| 202843_at | DNAJB9 | 5.84E-07 | -1.89348 |
| 225502_at | DOCK8 | 0.0011122 | -1.77308 |
| 218854_at | DSE | 0.00134482 | -1.75697 |
| 221773_at | ELK3 | 6.71E-07 | -1.77531 |
| 222646_s_at | ERO1L | 0.000176027 | -1.85066 |
| 218498_s_at | ERO1L | 3.07E-05 | -1.84758 |
| 229390_at | FAM26F | 0.00409905 | -1.75745 |
| 225734_at | FBXO22 | 8.05E-07 | -1.75456 |
| 204007_at | FCGR3B | 0.00338066 | -1.7828 |
| 233898_s_at | FGFR1OP2 | 5.58E-07 | -1.80289 |
| 227265_at | FGL2 | 6.42E-05 | -1.93117 |
| 203988_s_at | FUT8 | 2.56E-06 | -2.00595 |
| 205890_s_at | GABBR1 /// UBD | 0.000433147 | -2.38637 |
| 201724_s_at | GALNT1 | 4.81E-06 | -1.8049 |
| 238756_at | GAS2L3 | 0.000100668 | -1.81197 |
| 202270_at | GBP1 | 0.000188763 | -2.01793 |
| 231577_s_at | GBP1 | 0.00105324 | -1.77853 |
| 232024_at | GIMAP2 | 0.000872061 | -1.77736 |
| 205488_at | GZMA | 0.00014183 | -2.0867 |
| 225297_at | HAUS1 | 7.38E-08 | -1.82549 |
| 228697_at | HINT3 | 0.00412929 | -1.92596 |
| 202557_at | HSPA13 | 8.02E-05 | -1.81863 |
| 208965_s_at | IFI16 | 0.000777373 | -1.77098 |
| 206332_s_at | IFI16 | 9.67E-05 | -1.75571 |
| 214453_s_at | IFI44 | 0.00364683 | -1.78752 |
| 204415_at | IFI6 | 0.00385941 | -1.98368 |
| 229450_at | IFIT3 | 0.00121184 | -1.96725 |
| 206693_at | IL7 | 0.000185792 | -1.81806 |
| 202859_x_at | IL8 | 0.000801206 | -2.31049 |
| 222698_s_at | IMPACT | 9.21E-09 | -2.1685 |
| 218637_at | IMPACT | 1.58E-07 | -1.9318 |
| 217894_at | KCTD3 | 1.07E-06 | -1.80463 |
| 229850_at | KDSR | 1.38E-06 | -1.93273 |
| 1558279_a_at | KDSR | 1.08E-05 | -1.79319 |
| 226534_at | KITLG | 7.40E-06 | -1.8462 |
| 217388_s_at | KYNU | 5.64E-05 | -2.26288 |
| 218701_at | LACTB2 | 0.00010935 | -1.75318 |
| 213880_at | LGR5 | 0.00356176 | -2.67238 |
| 241607_at | LOC730102 | 0.00428868 | -1.76394 |
| 206584_at | LY96 | 5.88E-05 | -2.11143 |
| 1555745_a_at | LYZ | 0.000477335 | -4.07836 |
| 213975_s_at | LYZ | 0.000721143 | -1.89033 |
| 225160_x_at | MDM2 | 6.02E-06 | -1.89405 |
| 229711_s_at | MDM2 | 1.26E-05 | -1.78364 |
| 224725_at | MIB1 | 5.83E-05 | -1.86032 |
| 224720_at | MIB1 | 3.87E-05 | -1.75778 |
| 204580_at | MMP12 | 0.000284165 | -2.26718 |
| 204162_at | NDC80 | 8.86E-06 | -1.85885 |
| 226810_at | OGFRL1 | 5.97E-05 | -1.85241 |
| 219148_at | PBK | 0.00104853 | -1.95683 |
| 226452_at | PDK1 | 0.000114157 | -1.77671 |
| 225688_s_at | PHLDB2 | 0.00217506 | -1.85285 |
| 210145_at | PLA2G4A | 0.00144242 | -2.58431 |
| 213241_at | PLXNC1 | 0.00108721 | -1.80079 |
| 204286_s_at | PMAIP1 | 2.41E-05 | -1.96477 |
| 204285_s_at | PMAIP1 | 0.000110465 | -1.93898 |
| 209598_at | PNMA2 | 0.000278485 | -2.43892 |
| 204748_at | PTGS2 | 0.00120791 | -2.2952 |
| 212588_at | PTPRC | 0.000175817 | -1.96745 |
| 228708_at | RAB27B | 7.33E-05 | -2.64312 |
| 213982_s_at | RABGAP1L | 1.04E-08 | -1.76329 |
| 204070_at | RARRES3 | 0.00289131 | -1.83599 |
| 205590_at | RASGRP1 | 2.54E-05 | -1.76193 |
| 203344_s_at | RBBP8 | 1.60E-06 | -1.77849 |
| 225202_at | RHOBTB3 | 0.000567503 | -1.93838 |
| 235199_at | RNF125 | 0.00245966 | -1.80438 |
| 239143_x_at | RNF138 | 1.30E-07 | -1.77486 |
| 225541_at | RPL22L1 | 5.69E-07 | -2.48396 |
| 225953_at | RPRD1A | 1.45E-06 | -1.75702 |
| 213262_at | SACS | 0.000159156 | -1.85171 |
| 228653_at | SAMD5 | 0.00146913 | -2.43295 |
| 242626_at | SAMD5 | 0.00137079 | -2.30313 |
| 226603_at | SAMD9L | 0.00145733 | -1.78907 |
| 220330_s_at | SAMSN1 | 4.77E-05 | -1.99031 |
| 205352_at | SERPINI1 | 0.000172412 | -1.79134 |
| 212989_at | SGMS1 | 2.32E-05 | -1.75506 |
| 222838_at | SLAMF7 | 0.000516181 | -1.93411 |
| 232277_at | SLC28A3 | 0.000367502 | -2.24888 |
| 202088_at | SLC39A6 | 2.90E-08 | -1.75224 |
| 202527_s_at | SMAD4 | 1.33E-05 | -1.85357 |
| 212569_at | SMCHD1 | 5.28E-06 | -1.79771 |
| 227542_at | SOCS6 | 6.08E-06 | -1.82359 |
| 1566342_at | SOD2 | 6.32E-05 | -1.77182 |
| 202817_s_at | SS18 | 3.66E-06 | -2.01561 |
| 217790_s_at | SSR3 | 0.00013578 | -1.79882 |
| 205542_at | STEAP1 | 0.00100237 | -1.76219 |
| 230560_at | STXBP6 | 0.00210149 | -1.78162 |
| 208986_at | TCF12 | 6.64E-06 | -1.80339 |
| 205943_at | TDO2 | 0.00376235 | -2.00209 |
| 224793_s_at | TGFBR1 | 1.76E-05 | -1.82457 |
| 226117_at | TIFA | 5.47E-08 | -1.79069 |
| 219410_at | TMEM45A | 0.00146413 | -2.01775 |
| 206026_s_at | TNFAIP6 | 0.00291042 | -2.10577 |
| 210260_s_at | TNFAIP8 | 1.78E-06 | -2.01469 |
| 208296_x_at | TNFAIP8 | 1.48E-06 | -1.9402 |
| 210643_at | TNFSF11 | 0.00127014 | -1.86161 |
| 223502_s_at | TNFSF13B | 4.51E-05 | -2.09115 |
| 223501_at | TNFSF13B | 4.54E-05 | -2.0025 |
| 213293_s_at | TRIM22 | 0.00230209 | -1.75456 |
| 225406_at | TWSG1 | 1.74E-05 | -1.83641 |
| 222731_at | ZDHHC2 | 0.000532781 | -2.1027 |
